# Interleaved T2 preparation for simultaneous coronary artery and pulmonary artery and vein visualization

**DOI:** 10.1186/1532-429X-15-S1-E4

**Published:** 2013-01-30

**Authors:** Britta Butzbach, Marcelo Andia-Kohnenkampf, Markus Henningsson, Tarique Muhammad Hussain, Israel Valverde, Dirk Lossnitzer, Rene M Botnar, Gerald F Greil

**Affiliations:** 1 Imaging Sciences, King's College London, London, UK

## Background

**Figure 1:**
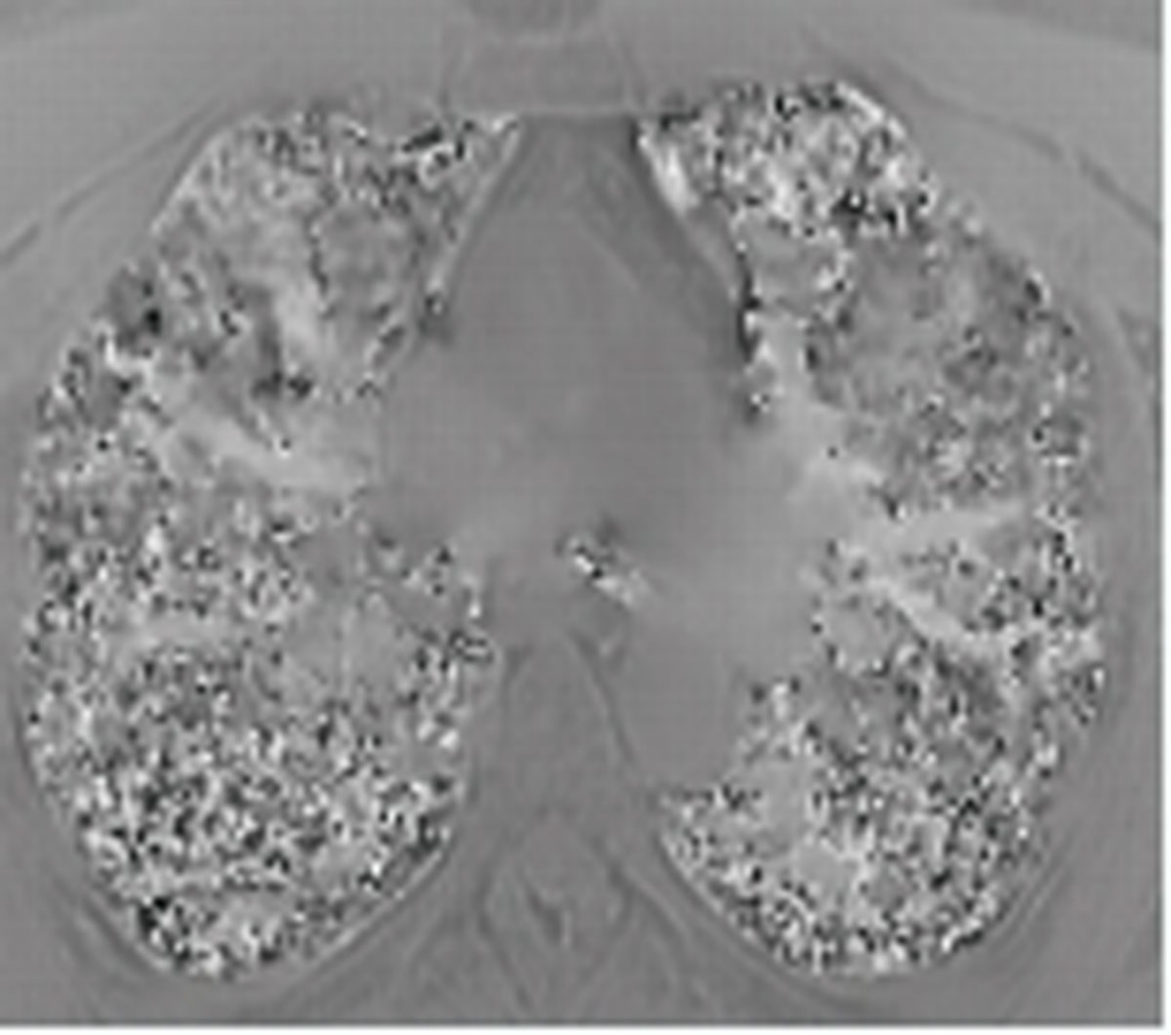
B0-map of a healthy volunteer, which shows the off-resonance phenomenon (bright signal: pulmonary veins).

Evaluation of a dynamic T2-preparation (dynT2prep) sequence, whereby the first dynamic is acquired with and the second without T2 preparation. T2 preparation allows suppression of tissues with short T2 relaxation times, e.g. deoxygenated blood. Tissues with long T2 relaxation times, e.g. oxygenated arterial blood, are minimally influenced. Thus both blood of the pulmonary veins and left ventricle should appear signal suppressed on subtracted dynT2prep images (T2prep OFF - T2prep ON). Subtraction of both dynamics, however, always resulted in black blood images of the great cardiac chambers and coronary arteries while the pulmonary circulation appeared bright due to a frequency offset in the lungs (Figure 1). This method was then tested in children with congenital heart disease (CHD) and healthy adult volunteers for selective pulmonary circulation visualization.

**Figure 2:**
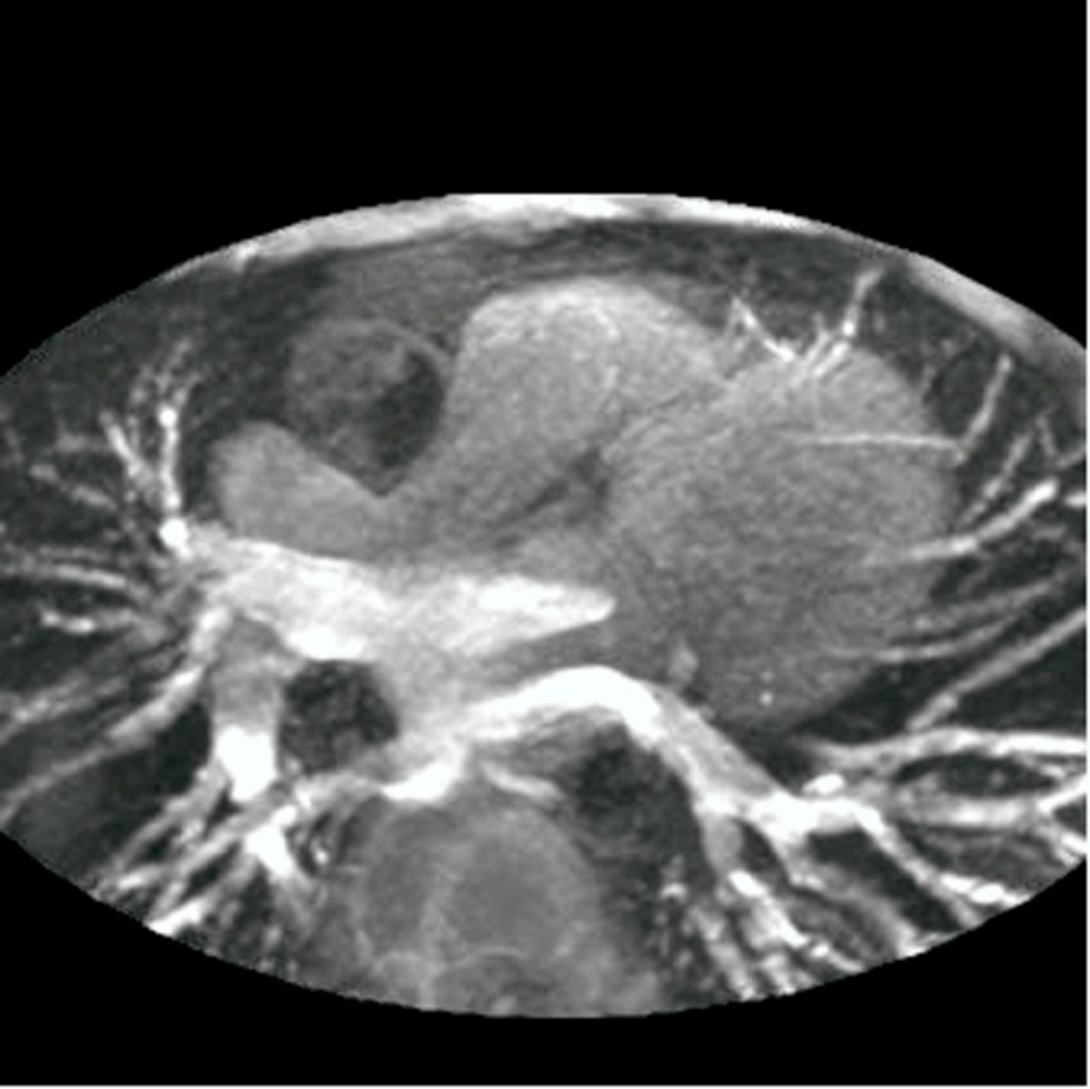
Pulmonary vein presentation in a healthy volunteer (bright signal)

## Methods

Patients with congenital heart disease (CHD) were examined with a dynamic T2prep 3D-whole heart acquisition (mean age 3 +/- 6.4 years, n=4) and compared to ten healthy subjects (mean 34 years, STD +/- 6.87, n=10) in diastole. An independent samples t-test and a paired t-test were performed to compare normally distributed variables.

## Results

An increase of SNR was seen in children and healthy volunteers in the subtracted images. After analyzing the B0-map, statistically significant differences were shown for the pulmonary veins (p<0.027).

## Conclusions

The brightest signal in all cases was observed in the pulmonary circulation. This effect is caused by an off-resonance phenomenon. Whilst using T2-preparations pulses, the B0-field inhomogeneity acts as a susceptibility background gradient and leads to a suppression of moving tissue and blood and consequently to a signal enhanced pulmonary circulation in subtraction images.

## Funding

Division of Imaging Sciences, The Rayne's Insitute

King's College London

